# Sorptive process and breakthrough behavior of odorous volatile compounds on inert surfaces

**DOI:** 10.1038/s41598-018-31362-0

**Published:** 2018-09-03

**Authors:** Ezaz Ahmed, Jan E. Szulejko, Adedeji A. Adelodun, Satya Sundar Bhattacharya, Byong Hun Jeon, Sandeep Kumar, Ki-Hyun Kim

**Affiliations:** 1 Department of Civil and Environmental Engineering, Hanyang University, 222 Wangsimni-Ro, Seoul, 04763 Korea; 2 Department of Marine Science and Technology, School of Earth and Mineral Science, The Federal University of Technology, P.M.B. 704 Akure, Nigeria; 3 Department of Environmental Science, Tezpur University, Tezpur, Assam 784028 India; 4 Department of Natural Resources and Environmental Engineering, Hanyang University, Seoul, 133-791 Korea; 5 Department of Bio and Nano Technology, Guru Jambheshwar University of Science and Technology, Hisar, Haryana 125001 India

## Abstract

The use of glass impinger is an important device for sampling and handling when measuring volatile organic compounds (SVOCs). Thus, it is important to check for possible analyte losses to the inner glass surface when carrying out sample analysis with the aid of impinger system. In this research, we evaluated the sorptive loss patterns of vapor-phase semi-volatile organic compounds [SVOCs (n = 10): acetic acid (ACA), propionic acid (PPA), i-butyric acid (IBA), n-butyric acid (BTA), i-valeric acid (IVA), n-valeric acid (VLA), phenol (PhAl), p-cresol (p-C), indole (ID), and skatole (SK)] on inert surfaces of an impinger in reference to sampling bags. The gaseous standard of these SVOCs (48–406 ppb) in polyester aluminum (PEA) bags was passed through an empty impinger in 1 L steps. The exiting SVOCs were collected on three-bed sorbent tubes for subsequent analysis by thermal desorption-gas chromatography-mass spectroscopy (TD-GC-MS). Impinger wall sorption capacities ranged from 2.0 to 21.0 ng cm^−2^. The 10% breakthrough adsorption capacities on the impinger wall for acids, phenols, and indoles ranged from 1.21 ± 0.15 to 5.39 ± 0.79, 0.92 ± 0.12 to 13.4 ± 2.25, and 4.47 ± 0.42 to 5.23 ± 0.35 ng cm^−2^, respectively. The observed sorptive patterns suggest that the sorptive losses of the volatile fatty acids, phenols, and indoles can occur very effectively at low ppb levels onto a glass surface.

## Introduction

Odor emissions from industrial and agricultural facilities are a common nuisance. A number of volatile organic compounds (VOCs) including volatile fatty acids (VFAs), phenols, indoles, and skatoles are generally designated as key target malodorants released from such sources^1–3^. High-level (acute) and low-level (chronic) exposures can have a negative impact on both human health and the environment^4^. For instance, in the case of VFAs, the most frequently reported health symptoms include irritation, headache, nausea, diarrhea, chest tightness, shortness of breath, and stress^5^. In addition to prominent health and environmental effects, some volatile organics are critically important in paleontology as formic and acetic acids outgassing from wooden storage cabinets were suggested to have deleterious effects on ancient glassware specimens held in museums^6^.

Phenols are also mildly toxic chemicals that are undesirable in biological systems as they are generally protoplasmic poisons and also corrosive to the eyes, skin, and respiratory tract^7^. p-Cresol is considered a prototypic protein-bound uremic toxin that can be responsible for kidney failure^8,9^. Phenol and p-cresol are both known as promoters of skin tumors and are associated with other epithelial tumors^10^. Indole and skatole are known to exhibit an intense fecal odor^11^. Skatole causes acute bovine pulmonary emphysema and toxic to human bronchial epithelial cell lines^11–13^. It is also used by the U.S. military in its non-lethal weaponry, specifically, malodorants.

The proper selection of the sampling and storage method is critically important to accurately quantify semi-volatile odorants. This is because the overall uncertainty of the measurements can be sensitively affected by the temporal stability of sample storage^14,15^. Whole air sampling by bags and canisters can be subjected to significant sample loss during storage, especially for semi-volatile organic compounds (SVOCs) like VFAs^6,14,16,17^. The use of a sorbent tube is another preferable option for sampling gaseous VOCs^18,19^. Although the use of stainless steel has been favored for such purposes, it can suffer significantly from sorptive analyte losses of SVOCs. To resolve such a problem, the use of glass, quartz, or inert (quartz)-coated steel sorbent tubes has been recommended. However, the sorptive losses of SVOCs to tube materials used to hold or pack sorbent is poorly documented^20^.

The use of glass impinger is an important choice for analyzing volatile organic compounds when employing headspace sampling approach. Particularly, it is necessary to check the possible losses of analyte due to the interactions with the inner glass surface during the sample treatment and/or analysis. Thus, it is crucial to define and characterize the performance metrics of each target species against glass walls, especially when the headspace analysis of SVOCs is carried out for their accurate, repeatable, and reliable quantitation. In this research, the sorptive losses of low ppb SVOCs on a relatively inert surface of a glass impinger were studied. To this end, a standard containing the 10 target odorant species (VFAs, phenols, and indoles) in nitrogen was pulled through an impinger. Therefore, it was equally important to know the recovery efficiency of the target species from polyester aluminum (PEA) bags used for (short-term) storage of standards prior to the use of standard gases for the impinger-based test. The sorptive loss patterns of these target VOCs were thus measured by monitoring the differences in their concentrations between the impinger’s inlet ([C]_in_) and outlet ([C]_out_). The results obtained from this study allowed us to examine the relative sorptive properties of each target on the glass surface (inner wall of the impinger) inferred by the mass balance.

## Results and Discussion

### Results of the calibration experiment and quality assurance

**Figure 1:**
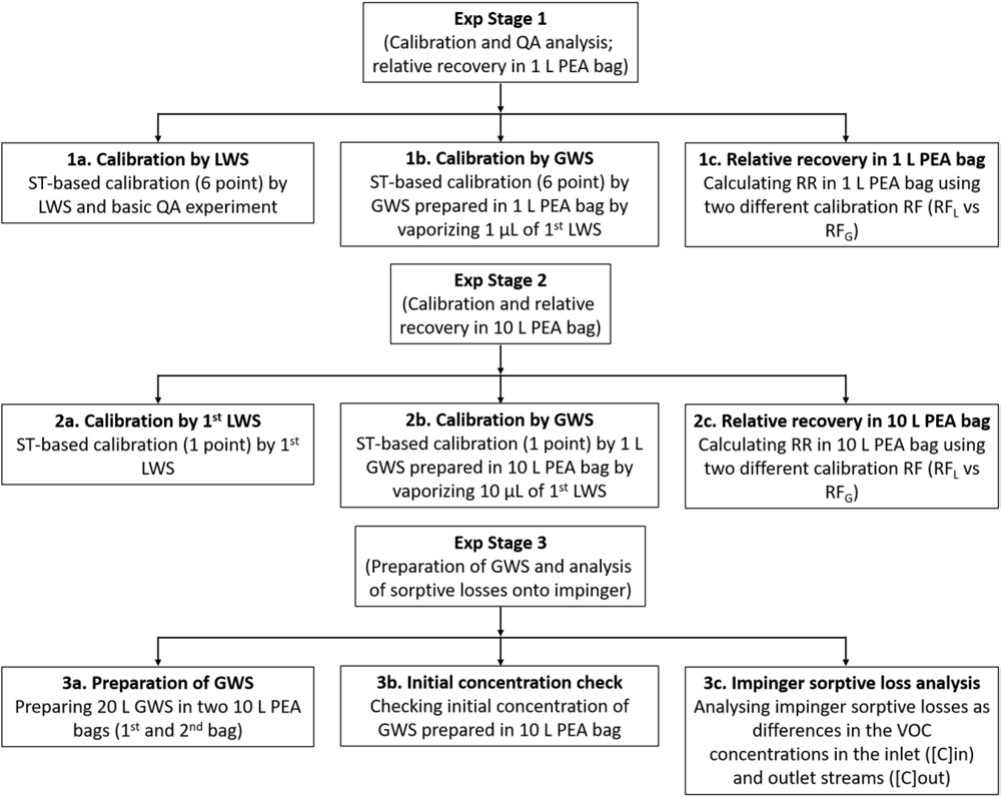
Basic flow chart for the experiments into sorptive losses of VFAs, phenols, and indoles on a glassware surface.

Table S1 presents the calibration results of both the LWS and GWS for a total of 10 target compounds [viz., 6 volatile fatty acids acetic acid (ACA), propionic acid (PPA), i-butyric acid (IBA), n-butyric acid (BTA), i-valeric acid (IVA), and n-valeric acid (VLA); phenol (PhAl), p-cresol (p-C), indole (ID), and skatole (SK)]. The response factor (RF) values obtained from the LWS- (Fig. 1; Exp. stage 1a) and GWS-based calibrations (Fig. 1; Exp. stage 1b) are denoted as RF_L_ and RF_G,_ respectively. The obtained RF_L_ values varied in a range from 5041 (PPA) to 34923 (SK). In general, the RF values of the low molecular weight VFAs were lower [e.g., ACA (5313) and PPA (5041)], while those of the high molecular weight VOCs exhibited relatively higher values [such as ID (28460) and SK (34923)]. However, the relatively low responses of the 1^st^ stage calibration by LWS for the lower molecular weight VOCs (e.g., ACA and PPA) were due to a combination of cold trap (CT) and sorbent tube (ST) breakthrough and the fraction of the electron impact ionization (EI) in the total ion current (TIC) chromatogram mass window^21^. The response factor values of VFAs (Exp. Stage 1b) using the GWS were also similar to the earlier (Exp. Stage 1a) calibration patterns (viz., dependence of the RF values on the molecular weights). However, the results of the phenolic and indolic compounds were much lower, as they were subject to higher sorptive losses in the 1 L PEA bag. The representative chromatograms obtained by the thermal desorption-gas chromatography-mass spectrometry (TD-GC-MS) system of the 5^th^ calibration point LWS and GWS (same loaded mass for both standards) (Fig. S1) also indicates a considerable peak intensity differences for phenol, p-cresol, indole, and skatole between the two calibration approaches.

As part of the basic quality assurance, the detection properties of the ST-TD-GC-MS system were evaluated in terms of the method detection limits (MDL) and reproducibility [relative standard error (RSE)] by using the LWS used for the first stage calibration (Table S1). To this end, experiments were conducted by loading 1 μL of LWS [the first concentration level (C1)] on a three-bed ST. The MDL values were calculated as the product of the SD and the Student’s *t*-value (3.14) at 99% confidence level. The MDL derived in terms of the absolute mass ranged from 0.62 to 2.59 ng for SK and ACA, respectively. The DL values corresponding to the mixing ratios (ppb) were derived by assuming a 1 L sample volume such as (0.12 to 1.08) ppb at 1 bar, respectively. The reproducibility of calibration, if derived in terms of RSE (%), was found in a range of 0.23 (ID) to 4.06 (VLA).

### General patterns of relative recovery of VFAs, phenols, and indoles in PEA bags

Vaporization is a common approach to transform liquid-phase standards into gas-phase standards. The relative recovery between the two phases is influenced by a number of factors (viz., temperature, flow rate of purge gas, physical properties of compounds, sorption on surfaces, etc.). The GWS produced via vaporization is often stored in a sampling bag. However, sorptive losses of SVOCs may occur on the bag’s inner surface, as it can be affected by bag material type and storage time^14,17,22,23^.

In order to accurately assess the relative recovery of SVOCs from PEA bags, the RR_PEA_ values were computed using the formula presented in Eqn. 1. Table S2 shows the results of the relative recovery (RR_PEA_) test of all target compounds in 1 and 10 L PEA bags. Accordingly, in the 1 L PEA bag, the RR values of the VFAs (78–108% for 6 VFAs) were generally higher than those of the phenols (41.9 and 47.6% for PhAl and p-C) and indoles (14.8 and 13.7% for ID and SK, respectively). As such, the RR values of the VFAs generally decreased as a function of their molecular weight (Table 1). The considerably reduced RR values of the phenols and indoles can be attributed to their high reactivity towards surface materials^22,24,25^. The RR_PEA_ values for the 10 L PEA bag (Table S2) also indicated similar high values for VFAs relative to phenols and indoles. In fact, the extent of RR in the 10 L PEA bag was slightly higher than that of the 1 L PEA bag for most of the target species (except for IBA and IVA). From this observation, the comparatively high recovery for the 10 L PEA bag is likely to reflect the effect of its smaller surface-to volume (S/V) ratio of (S/V = 0.32 cm^−1^) relative to a 1 L PEA bag (S/V = 0.72 cm^−1^)^23^.

**Table 1:** List of 10 target compounds (6 VFAs, 2 phenols, and 2 indoles) investigated to assess soprtive behavior on glass surfaces.

Full Name	Short Name	Chemical Formula	CAS Number	Molecular weight (g mol^−1^)	Purity (%)	Density (g mL^−1^)	Quantification ions (m/z)	Carbon Number
Acetic acid	ACA	C_2_H_4_O_2_	64-19-7	60.1	99.9	1.05	43, 45, 60	2
Propionic Acid	PPA	C_3_H_6_O_2_	79-09-04	74.1	99.0	0.99	73, 74	3
i-Butyric Acid	IBA	C_4_H_8_O_2_	79-31-2	88.1	99.0	0.97	73	4
n-Butyric Acid	BTA	C_4_H_8_O_2_	107-92-6	88.1	99.0	0.96	60	4
i-Valeric Acid	IVA	C_5_H_10_O_2_	503-74-2	102	99.0	0.93	60	5
n-Valeric Acid	VLA	C_5_H_10_O_2_	109-52-4	102	99.0	0.94	60	5
Phenol	PhAl	C_6_H_6_O	108-95-2	94	99.0	1.07	94	6
p-Cresol	p-C	C_7_H_8_O	106-44-5	108	99.7	1.03	107, 108	7
Indole	ID	C_8_H_7_N	120-72-9	117	99.0	1.17	117	8
Skatole	SK	C_9_H_9_N	83-34-1	131	98.0	1.10	130, 131	9

Table 2 presents a comparison of the relative recovery (RR) of the target species between this work and the literature. Trabue *et al*.^17^ observed noticeably high RR for ACA (273%) and PhAl (2793%) from the Tedlar bag due to surface outgassing^26,27^. If the recovery results are compared between the different studies, one can readily find the effects of the bag material and storage time on such quantitation^14,17^. Batterman *et al*.^28^ also investigated the recovery of some VOCs using electro-polished stainless-steel canisters and noticed a considerable decrease in the first hour, whereas the losses continued over a 16 day period at a slower rate. The Henry’s law constant (HLC) is positively correlated to the affinity of the odorants on surfaces. Hence, to learn more about the recovery patterns, the HLC (pmol cm^−2^ Pa^−1^) of the saturated inner surface of PEA bags (1 and 10) L and the impinger was computed for comparisons with other studies (Table 3). As expected, we confirmed the extent of sample losses was thus confirmed to be dependent on the sorptive properties of the surface materials^14,17,29^.

**Table 2:** Comparison of the relative recovery (%) of odorants with other studies for various sampling bags.

Bag type^a^ (Size)^b^	Storage time (hr)	ACA	PPA	IBA	BTA	IVA	VLA	PhAl	p-C	ID	SK	Reference
PEA (1 L) (n = 3)	<0.25	82.5 ± 5.14	88.1 ± 3.38	100.6 ± 4.02	95.1 ± 0.16	108.2 ± 0.78	93.1 ± 1.38	44.0 ± 2.98	43.8 ± 5.32	14.5 ± 0.52	13.6 ± 0.10	This study
PEA (10 L) (n = 3)	<0.25	87.7 ± 2.98	90.2 ± 3.11	97.5 ± 2.15	97.3 ± 2.18	100.1 ± 0.21	95.2 ± 0.30	65.8 ± 4.99	72.0 ± 3.81	29.9 ± 4.51	26.3 ± 3.44	This study
PET (Melinex)	0.5	84.8	88.0	105	81.9	88.6	67.6	—^c^	36.0	0.0	—	^14^
	24	27.6	61.4	109	73.9	102	51.1	—	5.6	0.0	—	^14^
PEP (Teflon)	0.5	101	100	96.5	88.2	85.5	73.8	—	66.9	38.2	—	^14^
	24	45.4	65.8	67.8	53.8	61.8	24.9	—	28.6	24.7	—	^14^
In-house PVF (Tedlar)	0.5	68.6	85.5	84.6	70.1	74.3	62.7	—	13.3	8.5	—	^14^
	24	23.0	53.5	79.8	44.5	61.6	24.8	—	22.7	23.2	—	^14^
Commercial PVF (Tedlar)	0.5	72.9	83.1	86.2	84.7	86.2	72.3	—	16.0	67.4	—	^14^
	24	20.4	43.2	57.1	37.4	52.6	20.6	—	3.1	0.0	—	^14^
Foil (LDPE)	0.5	29.7	0.0	47.9	0.0	35.0	12.8	—	2.7	19.6	—	^14^
	24	0.0	0.0	23.0	6.20	12.9	0.0	—	3.7	0.0	—	^14^
Polyester bag	2	—	—	35.0	40.0	39.0	—	—	—	6.5	13.0	^50^
Tedlar bag	24	273	72.0	84.8	68.5	62.7	50.3	2793	5.4	0.0	0.0	^17^

^a^PEA, PET, PEP, and PVF denote polyester aluminium, polyethylene terephthalate, fluorinated ethylene propylene copolymer, and polyvinyl fluoride (Tedlar)^®^, respectively.

^b^Bags are 10 L unless stated otherwise.

^c^Not tested.

**Table 3:** Comparison of the surface Henry’s law constant (HLC) of the odorants in different studies.

Surface	HLC (pmol cm^−2^ Pa^−1^)											GWS MeOHconc. & pressure	Surface area	References
	MeOH	ACA	PPA	IBA	BTA	IVA	VLA	PhAl	p-C	ID	SK			
1 L PEA bag	**—** ^**a**^	61.1	30.9	3.63	8.17	−10	11.5	202	140	673	662	0.792 mg L^−1^	720 cm^2^	This study
PP (Pa)	—	0.092	0.026	0.038	0.037	0.05	0.043	0.017	0.093	0.015	0.008	61000		
10 L PEA bag	—	59.1	54.1	12.1	14.4	0.0	14.4	184	121	684	728	0.792 mg L^−1^	3225 cm^2^	
PP (Pa)	—	0.105	0.026	0.038	0.037	0.047	0.045	0.026	0.136	0.028	0.015	61000		
173 mL impinger^**b**^ (n = 1)	—	3527	5061	4779	8593	14020	18147	3294	3204	7504	15359	0.792 mg L^−1^	325 cm^2^	
PP_(range)_ (mPa)	—	33.0–44.0	6.0–10.0	5.0–12.0	4.0–11.0	1.0–11.0	1.0–11.0	4.0–9.0	18.0–33.0	5.0–11.0	1.0–5.0	61000		
173 mL impinger^**c**^ (n = 3)	—	4180 ± 92	5784 ± 143	4990 ± 131	7325 ± 439	7863 ± 258	11794 ± 25	2810 ± 86	5222 ± 232	6232 ± 201	9083 ± 216	0.792 mg L^−1^	325 cm^2^	
PP_(range)_ (mPa)	—	36.0–41.0	4.0–5.0	4.0–5.0	3.0–5.0	4.0–6.0	3.0–5.0	4.0–6.0	69.0–78.0	7.0–10.0	5.0–6.0	61000		
25 mL vial	—	—	42.9	57.3	87.6	134.0	222.0	—	—	—	—	31.7 mg L^−1^	55 cm^2^	^29^
PP_(range)_ (Pa)	—	—	12.3–45.7	9.1–27.8	6.5–19.7	3.9–11.0	2.5–7.3	—	—	—	—	2400		
SiO_2_ (Knudsen cell)	43	1455	—	—	—	—	—	—	—	—	—	n/a	225 m^2^ g^−1^ (BET)	^39^
PP (mPa)	0.26	0.79	—	—	—	—	—	—	—	—	—			
α-Al_2_O_3_ (Knudsen cell)	1060	165669	—	—	—	—	—	—	—	—	—	n/a	12.8 m^2^ g^−1^ (BET)	^39^
PP (mPa)	0.26	0.79	—	—	—	—	—	—	—	—	—			

*PP indicates the “partial pressure”; Surface HLC was computed as the total adsorbed mol/surface area/VOC partial pressure.

^a^Not measured; ^b^Cleaning method A; ^c^Cleaning method B.

### Trends in the sorptive losses onto the impinger glass wall

The sorption of an organic species is highly dependent on the physicochemical properties of both the sorbent and target molecule^30^. The sorptive losses are controlled by the activation energy of the adsorption and mass transfer rates (molecular diffusion) that occur between the molecules and the surface^31^. Most sorption reactions tend to occur due to the physical attraction of the sorbent on a surface or by partitioning (dissolution) into different phases such as natural organic matter (NOM)^32^. However, sorption is still one of the most preferable options recommended for the removal of both organic and inorganic pollutants^33–35^.

**Figure 2:**
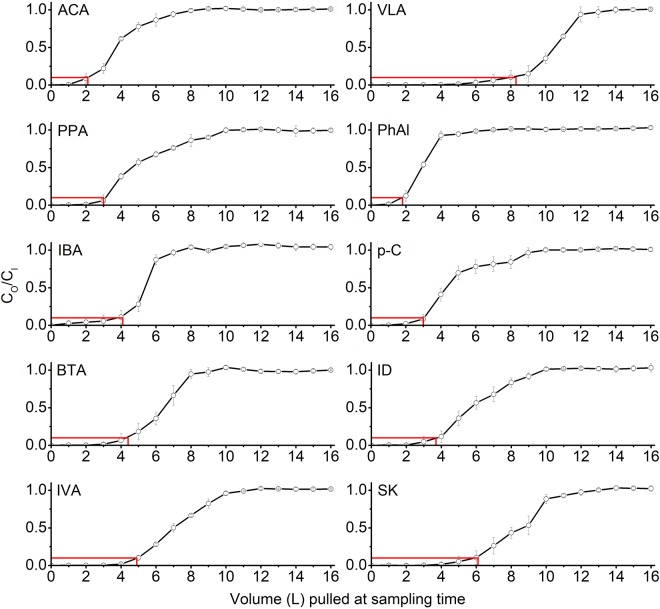
Normalized concentration values required for impinger glass saturation by GWS (normalization was made by [C_out_]/[C_in_]) (the error bars denote SD; the red lines indicate 10% BTV).

To investigate the sorptive loss trends of VOCs on an apparently “inert” glass surface, 16 L of the GWS was pulled through the impinger to observe the losses of the target SVOCs on the impinger glass surface. It was observed that the sorptive saturation for all targets was attained within a GWS volume of 16 L (equivalent to mass range of all target species at 4.70–30.5 µg) loaded onto the impinger. To understand more about the impinger saturation by target species, the [C_out_]/[C_in_] ratios vs. the loaded GWS volume were plotted (Fig. 2). The results indicate that all the target compounds attained sorptive saturation at different GWS loadings. A few VFAs (ACA, IBA, and BTA) attained sorptive saturation more rapidly (before 10 L) than others (PPA-11 L, IVA-12 L, and VLA-14 L). PhAl saturation seemed to occur at an earlier volume (7 L) than p-C (10 L). It is noted that ID reached saturation earlier at a 10 L sampling volume than SK (13 L). However, the sorptive saturation of 10 targets (ACA, PPA, IBA, BTA, IVA, VLA, PhAl, p-C, ID, and SK) was achieved after (3.07 to 19.1) µg were loaded onto the impinger. [The [C_out_]/[C_in_] ratios vs. the loaded GWS volume for the initial experiment using impinger (cleaning method A) is presented in Fig. S2].

Another means to meaningfully explain the sorptive capacity of a material is the breakthrough volume (BTV). To this end, the 10% BTV (i.e., 100 × [C_out_]/[C_in_] = 10) along with 5 and 50% BTV were arbitrarily chosen as reference points to evaluate the sorptive behavior of the target compounds^36^. Hence, the dynamic sorption capacity of the glass wall was computed at all three BTV values of 5, 10, and 50% BTV, as shown in Table 4. Most of the target compounds attained 5 and 10% breakthroughs within the first 5 L GWS loading volume [except VLA (6.8 and 8.6 L for both 5 and 10% BTV), respectively]. However, a BTV of PhAl (at 5% and 10%) was estimated at 1.5 and 1.8 L, while the 5% BTV for p-C was 2.6 L. Furthermore, 50% BTV increased with increasing VFA, phenol, and indole molecular weight (Table 4A) (e.g., 2.9 L for PhAl and 4.3 L for p-C and 5.7 and 8.7 L for ID and SK, respectively). Our study indicates that the trends in the sorptive losses of the target compounds onto the impinger glass surface increased with the molecular weight of the VFAs, phenols, and indoles^33^.

**Table 4:** Evaluation of the breakthrough volume (BTV), adsorbed mass, and adsorption capacity of the target compounds onto impinger glass at 5, 10, and 50% breakthroughs of the target compounds.

Target compounds	(A) BTV (L)			(B) Adsorbed mass (µg)			(C) Adsorption capacity (ng cm^−2^)^a^		
	5%	10%	50%	5%	10%	50%	5%	10%	50%
ACA	1.7 ± 0.46	2.1 ± 0.46	3.8 ± 0.15	1.45 ± 0.06	1.78 ± 0.07	2.67 ± 0.05	4.36 ± 0.19	5.39 ± 0.79	8.14 ± 0.10
PPA	2.7 ± 0.55	3.0 ± 0.38	4.7 ± 0.12	0.36 ± 0.01	0.40 ± 0.01	0.52 ± 0.02	0.98 ± 0.45	1.21 ± 0.15	1.60 ± 0.08
IBA	3.6 ± 0.29	4.1 ± 0.06	5.3 ± 0.23	0.49 ± 0.04	0.55 ± 0.04	0.65 ± 0.04	1.52 ± 0.22	1.77 ± 0.07	1.99 ± 0.13
BTA	3.9 ± 0.72	4.4 ± 0.78	6.5 ± 0.40	0.54 ± 0.06	0.60 ± 0.07	0.78 ± 0.11	1.68 ± 0.38	1.85 ± 0.42	2.41 ± 0.37
IVA	4.5 ± 0.29	4.9 ± 0.23	7.0 ± 0.21	0.89 ± 0.06	0.96 ± 0.07	1.23 ± 0.10	2.73 ± 0.36	2.98 ± 0.34	3.78 ± 0.35
VLA	6.8 ± 0.26	8.6 ± 0.45	10.5 ± 0.15	1.14 ± 0.06	1.42 ± 0.10	1.61 ± 0.12	4.02 ± 0.67	4.38 ± 0.41	4.96 ± 0.39
PhAl	1.5 ± 0.10	1.8 ± 0.26	2.9 ± 0.06	0.25 ± 0.01	0.30 ± 0.01	0.41 ± 0.01	0.80 ± 0.18	0.92 ± 0.12	1.26 ± 0.12
p-C	2.6 ± 0.41	3.0 ± 0.40	4.3 ± 0.31	3.64 ± 0.59	4.02 ± 0.68	5.24 ± 0.98	12.1 ± 2.71	13.4 ± 2.25	17.0 ± 4.42
ID	3.2 ± 0.72	3.7 ± 0.78	5.7 ± 0.46	1.26 ± 0.09	1.44 ± 0.10	1.93 ± 0.16	3.97 ± 0.40	4.47 ± 0.42	5.97 ± 1.04
SK	5.6 ± 0.64	6.1 ± 0.49	8.7 ± 0.69	1.64 ± 0.12	1.77 ± 0.14	2.23 ± 0.24	4.90 ± 0.28	5.23 ± 0.35	6.90 ± 0.94

^a^Adsorption capacity was computed as the adsorbed mass/inner surface of the glass wall.

### Adsorption capacity of the glass on the odorant SVOCs

The adsorption capacity is the amount of adsorbate taken up by the adsorbent per unit mass (or area) of a sorbent (or a surface), respectively. It can be used meaningfully to measure the adsorption performance of a material (or surface)^37^. The Henry’s law constant (HLC) is an important parameter for determining the adsorbate–adsorbent interactions, which characterize the adsorbent heterogeneity and adsorption affinity of the odorants^38^. Therefore, to learn about sorptive patterns, the HLC of the different odorants on the various surfaces used in our study were computed. Table 3 shows a comparison of the odorant HLCs from this study compared to previous studies. The ACA HLC (pmol cm^−2^ Pa^−1^) on the impinger glass (3527 and 4180 ± 92 for cleaning method A and B, respectively) (this work) is comparable to SiO_2_ (1455) but is much smaller than α-Al_2_O_3_ (165669)^39^. As expected, a significant difference in the HLC values of the VFAs between an impinger glass surface (173 mL) (this work) vs. a glass vial (25 mL) has been observed^29^. Notable differences in three different settings (such as the 173 mL impinger (cleaning method A), same impinger (method B), and 25 mL glass vial) were observed such as: PPA (5061, 5784 ± 143, and 42.9), IBA (4779, 4990 ± 131, and 57.3), BTA (8593, 7325 ± 439, and 87.6), IVA (14020, 7863 ± 258, and 134), and VLA (18147, 11794 ± 25, and 222) pmol cm^−2^ Pa^−1^, respectively. The higher impinger HLC values may be due to larger interactive surface area; and also the presence of silanol (SiOH) groups, which makes the surface adsorptive^40^.

**Figure 3:**
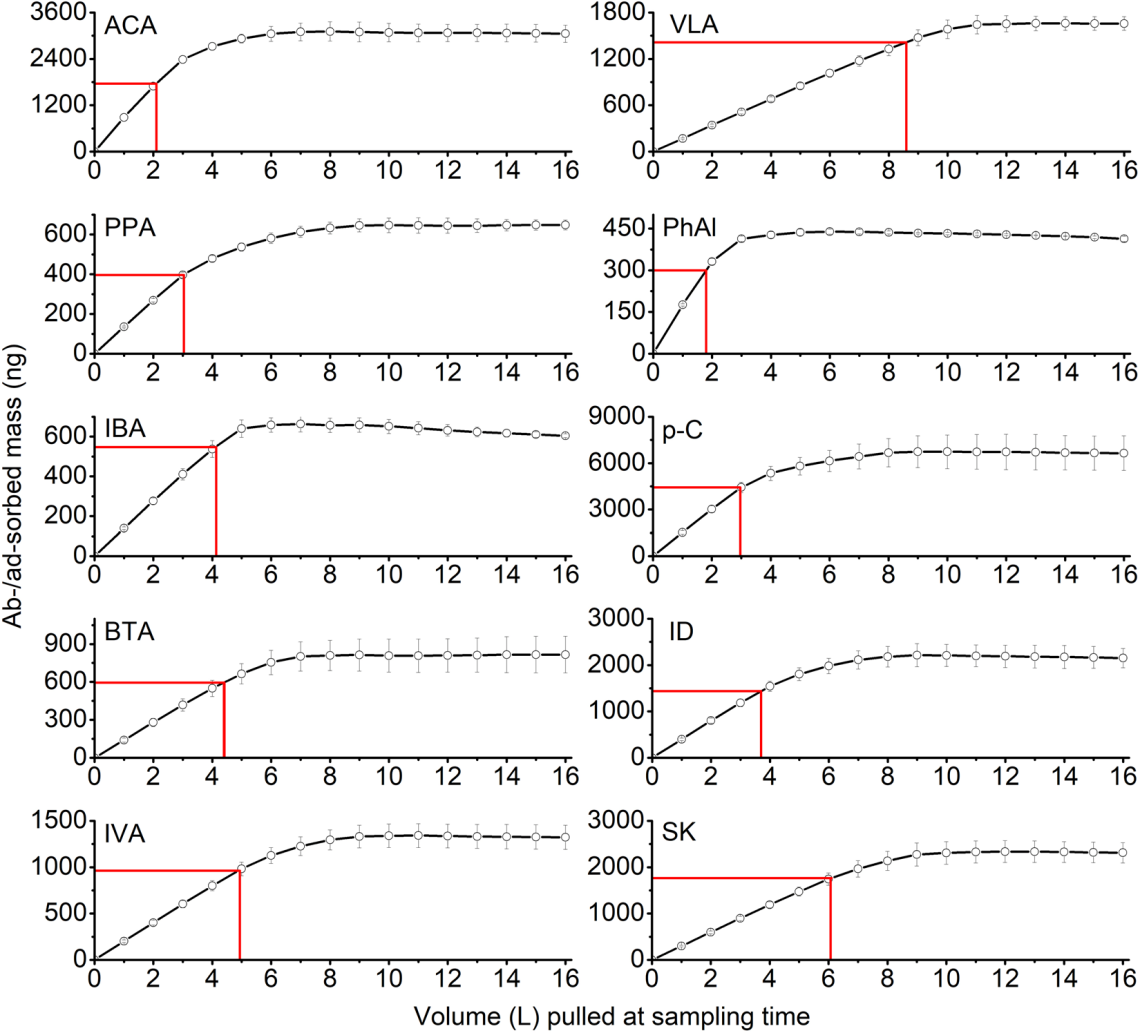
Analyte surface sorption (ng) vs. volume (L) pulled through the impinger (the error bars denote SD; the red lines indicate 10% BTV).

To determine the actual onset of the impinger saturation, we plotted the total mass (ng) adsorbed with respect to the volume (L) passed through the impinger. The adsorbed mass on the impinger glass wall is shown in Fig. 3 and Table 4B. It showed that the masses of six VFAs (ACA, PPA, IBA, BTA, IVA, and VLA) adsorbed onto the impinger glass wall (at 10% BTV) were computed as: (1.78 ± 0.07, 0.40 ± 0.01, 0.55 ± 0.04, 0.60 ± 0.07, 0.96 ± 0.07, and 1.42 ± 0.10) µg, respectively. Our study indicates a common trend (except for ACA) where heavier VFAs are more strongly adsorbed onto the impinger glass wall compared to their lighter counterparts. This could be explained due to the higher sorptive reactivity and increased dynamic sorption partitioning of the heavier VFAs onto the inner surface of the impinger wall^29,41^. However, the lower adsorbed mass (at 10% BTV) of PhAl (0.30 ± 0.01 µg) compared to p-C (4.02 ± 0.68 µg) is assumed due to their steric effects on the adsorption kinetics onto the impinger glass surface^42,43^, whereas the slightly lower adsorption of ID (1.44 ± 0.10 µg) compared to SK (1.77 ± 0.14 µg) may be due to the competitive adsorption and the formation of an inclusion complex through host-guest interactions^44–46^. [Total mass (ng) vs. loaded GWS volume (L) passed through the impinger (cleaning method A) is presented in Fig. S3].

In order to accurately assess the adsorption capacity of the odorants by the inert glass surface, the adsorption capacity (ng cm^−2^) at 5, 10, and 50% BTV were calculated (Table 4C). The adsorption capacity of the target odorants onto the impinger glass surface increased with the molecular weight of the VFAs, phenols, and indoles^33^. Therefore, the adsorption capacity of the target odorants is dependent on the sorptive properties of the surface materials^14,17^. Although glass is widely thought of as an “inert” material, glass surfaces are, in fact, slightly acidic and highly adsorptive, due to the presence of silanol groups (SiOH). Silanization of glass surfaces is sometimes used to reduce sample losses by converting reactive surface-OH groups into silyl derivatives^40^. Theoretically, sample losses should not occur or at the least level on the deactivated glass surfaces. Further intensive research is hence desirable to provide detailed understanding on the interactions of VOCs with deactivated surfaces.

## Conclusions

The relative recovery of the VFAs in PEA bags was higher than those of the phenols and indoles. The sorptive losses of the SVOCs on glass were clearly distinguishable between the target compounds. The dynamic sorptive capacity of the SVOCs onto the glass surface at 10% BTV tended to increase (except for ACA) with increasing molecular weight [(5.39 ± 0.79, 1.21 ± 0.15, 1.77 ± 0.07, 1.85 ± 0.42, 2.98 ± 0.34, and 4.38 ± 0.41) ng cm^−2^ for ACA, PPA, IBA, BTA, IVA, and VLA, respectively), (0.92 ± 0.12 and 13.4 ± 2.25) ng cm^−2^ for PhAl and p-C, respectively), and (4.47 ± 0.42 and 5.23 ± 0.35) ng cm^−2^ for ID and SK, respectively)].

Based on the relative sorptive patterns of SVOCs observed in this study, it is suggested that their inter-compound relationship should be tightly controlled in association with their respective physico-chemical properties. The significant sorptive losses of SVOCs (e.g, VFAs, phenols, and indoles) on the glass surface of an impinger took place very effectively and in a predictable manner. This study should thus assist in establishing the standard guide for accurate quantification of some important SVOCs in relation to storage media used for the actual measurements.

## Materials and Methods

### Preparation of sorbent tubes and liquid working standards

In this study, three-bed sorbent tubes (STs) were employed for sampling the SVOC standard gas masses (or concentration) entering (C_in_) and exiting the impinger (C_out_). Through quantification of the target odorants (i.e., C_in_ and C_out_), the extent of sorptive losses (by mass balance) occurring onto the inner glass wall surface of the impinger were estimated. The detailed procedure for preparing these STs has been presented elsewhere^47^. To prepare the STs, quartz tubes were packed with three types of sorbents (50 mg of each) in the following order (from weakest to strongest in the direction of the sample flow): Carbopack C (60/80 mesh), Carbopack B (60/80 mesh), and Carbopack X (40/60 mesh). In order to analyze a wide range of volatile and semi-volatile organic compounds, the three sorbents were chosen based on their physico-chemical properties^48^. The sorbents were held separately in place with the aid of quartz wool. All the sorbents were purchased from Supelco, USA. Before use, the STs were conditioned for 6 h at 320 °C by passing 99.99% N_2_ (flow rate = 100 mL min^−1^) with the aid of a tube conditioner (ATC-1200, ACEN Co. Ltd., Korea).

A total of 10 compounds (e.g., acetic acid (ACA), propionic acid (PPA), i-butyric acid (IBA), n-butyric acid (BTA), i-valeric acid (IVA), n-valeric acid (VLA), phenol (PhAl), p-cresol (p-C), indole (ID), and skatole (SK)) were selected to assess the sorptive properties on an impinger’s glass surface (Table 1). For this test, standards were prepared in a stepwise manner as follows: A series of liquid working standards (LWS) were prepared in methanol by diluting the laboratory grade chemicals of the target compounds (all purchased from Sigma–Aldrich, USA). The 1^st^ gaseous working standard (GWS) was obtained by vaporizing the 1^st^ LWS into a 1 L PEA bag. All reagents were used without further purification. Note that PhAl, p-C, ID, and SK are solids, while the others are liquids. Hence, the solid reagent grade chemicals were dissolved in methanol to make a 20 mL solution. Different aliquots of the reagents were then used to prepare LWS for a 6-point calibration of the TD-GC-MS (Table S3).

To obtain the calibration curves for all the target compounds, 1 µL of the LWS was directly injected (by a 10 μL syringe, SGE Analytical Science, Australia) into the ST (via piercing a 2 cm length of silicone tubing) into a constant stream of ultra-pure (99.99%) back-up N_2_ gas. (To this end, silicone tubing was used, as it is more convenient for piercing and handling relative to the Teflon tubing.) However, it should be noted that silicone is a strong sorbent for volatile organic compounds^49^. Therefore, the tubing (2 cm) was used carefully so that the analytes might not contact on the tubing wall. This back-up gas was passed through the ST at a constant flow rate of 100 mL min^−1^ (by a Sibata mini pump) for 3 min to facilitate adsorption of the analytes onto the sorbents. The STs were then analyzed by a thermal desorption–gas chromatography–mass spectrometry (TD–GC–MS) system as described below.

### Instrumental set-up

The analysis of all the target compounds was done using a TD unit (UNITY, Markes International Ltd., UK) interfaced to a GC (Shimadzu GC-2010, Japan) equipped with a mass spectrometric detector (Shimadzu GCMS-QP2010, Japan). The detailed operational condition of the TD-GC-MS system is described in Table S4. Upon collection of the sample, the ST was placed into the TD and heated to 300 °C (for 5 min) with a supply of ultrapure (99.999%) He to first desorb and then focus the targets in a cryofocusing trap (Carbopack C and Carbopack B) (CT) kept at 5 °C. The target compounds were then separated using a temperature programmed polar column (CP-WAX; Varian, USA). Please refer to Table S4 for further operational details of the TD-GC-MS.

### Experimental approaches

In order to assess the sorptive losses of the target compounds on the impinger glass surface, the GWS was prepared and temporarily stored in two 10 L PEA bags and then flowed through an empty impinger (173 mL volume; estimated 325 cm^2^ interior surface area; Schott Duran, Germany) at a constant flow (100 mL min^−1^) by means of a Sibata mini pump. It should be mentioned that the GWS prepared in each 10 L bag was stored for a maximum duration of 100 min (after preparation) to conduct impinger sorptive losses experiment. In an initial experiment, prior to use, an impinger was first cleaned with detergent and then distilled by water. It was finally air dried at 25 °C (cleaning method A). Subsequent experiments (triplicate) prior to each sorptive losses experiment, each 173 mL impinger (separate impinger for each experiment) was first cleaned by detergent, by distilled water, by methanol, and then finally dired at 110 °C in a closed oven (cleaning method B). [Please note that the sorptive loss experiments using impinger (by cleaning method B) is discussed throughout the manuscript unless it is specified.] Therefore, it was important to know the relative recovery (RR) efficiency of the target species in a 10 L PEA bag prior to the use of GWS for impinger-based analyte sorptive losses experiments. We also checked the RR of our targets in a 1 L PEA bag to check the surface to volume ratio effect on the recovery efficiency.

The basic experimental scheme of this study is depicted as a flow chart in Fig. 1. The first stage can be described as follows: at first, ST-based experiments were conducted by using 1 µL of each LWS for 6-point calibration and basic QA. Then, for the 1 L PEA bag recovery experiments, 1 µL of 1^st^ LWS was injected and vaporized into a 1 L PEA bag through the empty quartz tube (280 °C) under an ultrapure N_2_ flow (100 mL min^−1^ for 10 min). Finally, the relative recovery (RR) in a 1 L PEA bag was calculated by comparing the RF values derived with and without vaporization using the following formula:

1 $$RR\,\,( \% )=\frac{R{F}_{G}}{R{F}_{L}}\times 100$$

where, RF_G_ and RF_L_ indicate the response factor values obtained from the GWS and LWS, respectively.

**Figure 4:**
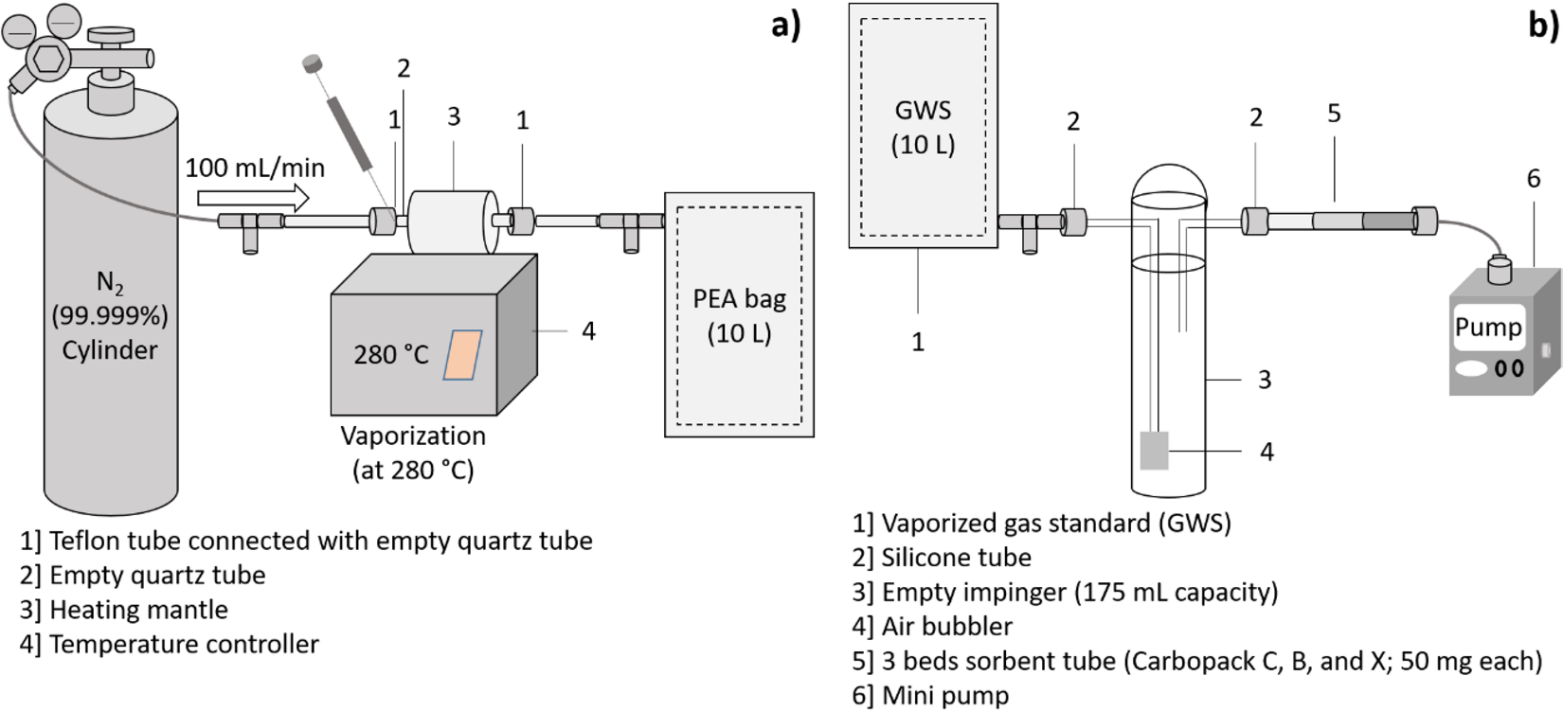
Schematic of the sorptive losses onto the impinge glass wall. Vaporization of the LWS to the gas standard (**a**) and test of VOC losses on the impinger by using GWS prepared by vaporizing liquid standard (**b**).

The second stage can be described as follows: at first, 1 µL of the 1^st^ LWS was directly injected into a three-bed sorbent tube for one-point RF calibration (RF_L_). Then, 10 µL of the 1^st^ LWS was injected and vaporized in an ultrapure N_2_ at a flow rate of 100 mL min^−1^ for 100 min into a 10 L PEA bag. This GWS was then used for ST-based one-point RF calibration (RF_G_). Finally, the RR was calculated as the ratios of the RF value between the vaporized standard and of the LWS (Eqn. 1). A schematic for the vaporization of the LWS into the GWS (in a 10 L) PEA bag is presented in Fig. 4a.

In the third stage, the sorptive loss of the target compounds on the impinger glass surface was measured with a near-continuous flow of vaporized (gas) standard (at 100 mL min^−1^ for 10 min) prepared in two 10 L PEA bags; the sample flow was only stopped after every 1 L for ~15 sec to replace the sampling ST with a fresh ST and to swop-out the PEA bag after a 8 L flow. The detailed experimental procedures are described as follows: At first, the target concentrations [C_in_] in the GWS were measured by loading onto an ST at a flow rate of 100 mL min^−1^ (see Fig. 4b). Then, the GWS was semi-continuously pulled through the impinger into STs. The concentration of each target exiting the impinger [C_out_] was measured by loading onto the three-bed STs at the same constant flow (100 mL min^−1^ for 10 min). The sorptive losses of the target compounds were then calculated from the differences in the VOC concentrations at the inlet ([C]_in_) and outlet streams ([C]_out_) using the formula:

2 $$Sorptive\,loss=({C}_{in}-{C}_{out})\times {\rm{\Delta }}V$$

where ∆V = 1 L, the volume sampled by the ST.

It should be noted that there was a short break (~30 sec) after 8 L of the GWS had been pulled through the impinger to exchange the other 10 L PEA bag for another 8 L supply of GWS.

## Electronic supplementary material

Supporting Information

## Data Availability

The data generated or analyzed during this study are included in either the article or in the Supplementary Information files.

## References

[1] Trabue S, Kerr B, Bearson B, Ziemer C (2011). Swine Odor Analyzed by Odor Panels and Chemical Techniques. J Environ Qual.

[2] Trabue S (2011). Identifying and tracking key odorants from cattle feedlots. Atmos Environ.

[3] Wright DW (2005). Multidimensional gas chromatography-olfactometry for the identification and prioritization of malodors from confined animal feeding operations. J Agr Food Chem.

[4] Sucker K, Both R, Winneke G (2009). Review of adverse health effects of odours in field studies. Water Science and Technology.

[5] Schiffman SS (2000). Potential health effects of odor from animal operations, wastewater treatment, and recycling of byproducts. Journal of Agromedicine.

[6] Robinet L, Hall C, Eremin K, Fearn S, Tate J (2009). Alteration of soda silicate glasses by organic pollutants in museums: Mechanisms and kinetics. J Non-Cryst Solids.

[7] Todorović V (2003). Acute phenol poisoning. Medicinski pregled.

[8] De Smet R (2003). Toxicity of free p-Cresol: A prospective and cross-sectional analysis. Clin Chem.

[9] Ketteler M (2006). Kidney failure and the gut: p-cresol and the dangers from within. Kidney Int.

[10] Boutwell RK, Bosch DK (1959). Tumor-Promoting Action of Phenol and Related Compounds for Mouse Skin. Cancer Res.

[11] Yasuhara A, Fuwa K (1977). Odor and Volatile Compounds in Liquid Swine Manure .2. Steam-Distillable Substances. B Chem Soc Jpn.

[12] Hammond AC, Carlson JR, Breeze RG (1980). Indole Toxicity in Cattle. Vet Rec.

[13] Nichols WK (2003). 3-methylindole-induced toxicity to human bronchial epithelial cell lines. Toxicol Sci.

[14] Koziel JA (2005). Evaluation of sample recovery of malodorous livestock gases from air sampling bags, solid-phase microextraction fibers, Tenax TA sorbent tubes, and sampling canisters. J Air Waste Manage.

[15] van Harreveld AP (2003). Odor concentration decay and stability in gas sampling bags. J Air Waste Manage.

[16] Beauchamp, J., Herbig, J., Gutmann, R. & Hansel, A. On the use of Tedlar (R) bags for breath-gas sampling and analysis. *J Breath Res* **2** (2008). 10.1088/1752-7155/2/4/046001 21386188

[17] Trabue SL, Anhalt JC, Zahn JA (2006). Bias of Tedlar bags in the measurement of agricultural odorants. J Environ Qual.

[18] Bates M (2008). Analysis of polycyclic aromatic hydrocarbons (PAHs) in airborne particles by direct sample introduction thermal desorption GC/MS. Atmos Environ.

[19] Woolfenden E (2010). Sorbent-based sampling methods for volatile and semi-volatile organic compounds in air. Part 2. Sorbent selection and other aspects of optimizing air monitoring methods. J Chromatogr A.

[20] Harper M (2000). Sorbent trapping of volatile organic compounds from air. J Chromatogr A.

[21] Szulejko JE, Kim YH, Kim KH (2013). Method to predict gas chromatographic response factors for the trace-level analysis of volatile organic compounds based on the effective carbon number concept. J Sep Sci.

[22] Kim YH, Kim KH (2015). Test on the Reliability of Gastight Syringes as Transfer/Storage Media for Gaseous VOC Analysis: The Extent of VOC Sorption between the Inner Needle and a Glass Wall Surface. Anal Chem.

[23] Kim YH (2012). Comparison of storage stability of odorous VOCs in polyester aluminum and polyvinyl fluoride Tedlar (R) bags. Anal Chim Acta.

[24] Gagarin S, Gyul’maliev A (2007). Heat of vaporization of methyl-substituted phenol derivatives. Coke and Chemistry.

[25] Keener KM, Zhang J, Bottcher RW, Munilla RD (2002). Evaluation of thermal desorption for the measurement of artificial swine odorants in the vapor phase. T Asae.

[26] Kushch, I. *et al*. Compounds enhanced in a mass spectrometric profile of smokers’ exhaled breath versus non-smokers as determined in a pilot study using PTR-MS. *J Breath Res* **2** (2008). 10.1088/1752-7155/2/2/026002 21383443

[27] Steeghs MML, Cristescu SM, Munnik P, Zanen P, Harren FJM (2007). An off-line breath sampling and analysis method suitable for large screening studies. Physiol Meas.

[28] Batterman SA, Zhang GZ, Baumann M (1998). Analysis and stability of aldehydes and terpenes in electropolished canisters. Atmos Environ.

[29] Kim YH, Kim KH, Szulejko JE, Parker D (2014). Development of the Detection Threshold Concept from a Close Look at Sorption Occurrence Inside a Glass Vial Based on the In-Vial Vaporization of Semivolatile Fatty Acids. Anal Chem.

[30] Delle Site A (2001). Factors affecting sorption of organic compounds in natural sorbent/water systems and sorption coefficients for selected pollutants. A review. J Phys Chem Ref Data.

[31] Jalaludin, Z. *The water vapour sorption behaviour of wood*, Edinburgh Napier University (2012).

[32] Adamson, A. W. & Gast, A. P. *Physical chemistry of surfaces*. (John Wiley & Sons, Inc., 1967).

[33] Kim YH, Kim KH (2012). Experimental approach to assess sorptive loss properties of volatile organic compounds in the sampling bag system. J Sep Sci.

[34] Rashed, M. N. *Chapter 7: Adsorption technique for the removal of organic pollutants from water and wastewater*. (InTech, 2013).

[35] Thompson A, Goyne K (2012). Introduction to the sorption of chemical constituents in soils. Nature Education Knowledge.

[36] Kim KH, Lee MH, Szulejko JE (2014). Simulation of the breakthrough behavior of volatile organic compounds against sorbent tube sampler as a function of concentration level and sampling volume. Anal Chim Acta.

[37] Mokhatab, S. & Poe, W. A. *Handbook of natural gas transmission and processing*. (Gulf professional publishing, 2012).

[38] Ruthven, D. M. *Principles of adsorption and adsorption processes*. (John Wiley & Sons, 1984).

[39] Carlos-Cuellar S (2003). Heterogeneous uptake kinetics of volatile organic compounds on oxide surfaces using a Knudsen cell reactor: Adsorption of acetic acid, formaldehyde, and methanol on alpha-Fe2O3, alpha-Al2O3, and SiO2. J Phys Chem A.

[40] Sandra P, Verzele M (1977). Surface Treatment, Deactivation and Coating in (GC)2 (Glass Capillary Gas-Chromatography). Chromatographia.

[41] Kim KH, Kim D (2009). A combination of Tedlar bag sampling and solid-phase microextraction for the analysis of trimethylamine in air: Relationship between concentration level and sample size. Microchem J.

[42] Liu QS, Zheng T, Wang P, Jiang JP, Li N (2010). Adsorption isotherm, kinetic and mechanism studies of some substituted phenols on activated carbon fibers. Chem Eng J.

[43] Singh BK, Rawat NS (1994). Comparative Sorption Kinetic-Studies of Phenolic-Compounds on Fly-Ash and Impregnated Fly-Ash. J Chem Technol Biot.

[44] El Mrabet S (2014). Competitive effect of the metallic canister and clay barrier on the sorption of Eu3+ under subcritical conditions. Appl Geochem.

[45] Gazpio C (2008). Sorption of pindolol and related compounds by a beta-cyclodextrin polymer: Isosteric heat of sorption. Carbohyd Polym.

[46] Geelhoed JS, Hiemstra T, VanRiemsdijk WH (1997). Phosphate and sulfate adsorption on goethite: Single anion and competitive adsorption. Geochim Cosmochim Ac.

[47] Iqbal MA, Kim KH, Szulejko JE, Cho J (2014). An assessment of the liquid-gas partitioning behavior of major wastewater odorants using two comparative experimental approaches: liquid sample-based vaporization vs. impinger-based dynamic headspace extraction into sorbent tubes. Anal Bioanal Chem.

[48] Wu CH, Feng CT, Lo YS, Lin TY, Lo JG (2004). Determination of volatile organic compounds in workplace air by multisorbent adsorption/thermal desorption-GUMS. Chemosphere.

[49] Sulyok M, Haberhauer-Troyer C, Rosenberg E (2002). Observation of sorptive losses of volatile sulfur compounds during natural gas sampling. J Chromatogr A.

[50] Nagata Y, Takeuchi N (2003). Measurement of odor threshold by triangle odor bag method. Odor Measurement Review.

