# Supplementary material for: Sorptive process and breakthrough behavior of odorous volatile compounds on inert surfaces
Source: Sci Rep. 2018 Sep 3;8:13118. doi: 10.1038/s41598-018-31362-0 (PMC6120927; doi:10.1038/s41598-018-31362-0)
Supplement: Supplementary file 1 — Supporting Information [file 41598_2018_31362_MOESM1_ESM.docx]

**Supporting Information**

**Sorptive process and breakthrough behavior of odorous volatile compounds on inert surfaces**

**Ezaz Ahmed^a^, Jan E. Szulejko^a^, Adedeji A. Adelodun^b^, Satya Sundar Bhattacharya^c^,**

**Byong Hun Jeon*^d^,** **Sandeep Kumar*^a,e^,** **Ki-Hyun Kim*^a^**

*^a^Department of Civil and Environmental Engineering, Hanyang University, 222 Wangsimni-Ro, Seoul 04763, Korea; ^b^Department of Marine Science and Technology, School of Earth and Mineral Science, The Federal University of Technology, P.M.B. 704, Akure, Nigeria; ^c^Department of Environmental Science, Tezpur University, Assam 784028, India; ^d^Department of Natural Resources and Environmental Engineering, Hanyang University, Seoul, 133-791, Korea; ^e^Department of Bio and Nano Technology, Guru Jambheshwar University of Science and Technology, Hisar-Haryana, 125001, India*

**Contents**

**Table 1S.** Results for the ST-based calibration of the liquid working standard (LWS) and vaporized gas standard (GWS) by TD/GC/MS

**Table 2S.** Relative recovery^a^ (RR_PEA_) of the target compounds in a 1 and 10 L PEA bag

**Table 3S.** Preparation of liquid working standards (LWS) for analysis by the TD/GC/MS system

**Table 4S.** Operational conditions of TD/GC/MS systems for comparative analysis of the target compounds

**Figure 1S.** The representative chromatograms of the target compounds obtained from the TD/GC/MS system by direct loading of 1 µL of the 5th calibration point LWS (3a) and 200 mL of vaporized GWS (3b) (same loading mass of LWS) onto the 3-bed sorbent tube: (elution order: (1) Acetic acid, (2) Propionic acid, (3) i-Butyric acid, (4) n-Butyric acid, (5) i-Valeric acid, (6) n-Valeric acid, (7) Phenol, (8) p-Cresol, (9) Indole, and (10) Skatole)

**Figure 2S.** Normalized concentration values required for 173 mL glass impinger (cleaned by method A) saturation by GWS (normalization was made by [C_out_]/[C_in_]) (the red lines indicate 10 % BTV)

**Figure 3S.** Analyte surface sorption (ng) vs. volume (L) pulled through the 173 mL glass impinger (cleaned by method A) (the red lines indicate 10 % BTV)

*Correspondence: [kkim61@hanyang.ac.kr](mailto:kkim61@hanyang.ac.kr); [ksandeep36@yahoo.com](mailto:ksandeep36@yahoo.com); [bhjeon@hanyang.ac.kr](mailto:bhjeon@hanyang.ac.kr)

**Table 1S.** Results for the ST-based calibration of the liquid working standard (LWS) and vaporized gas standard (GWS) by TD/GC/MS

| Compounds | ACA | PPA | IBA | BTA | IVA | VLA | PhAl | p-C | ID | SK |
| --- | --- | --- | --- | --- | --- | --- | --- | --- | --- | --- |
| (A) Comparison of the calibration curves derived by two types of STDs | | | | | |  |  |  |  |  |
| RF(L)^a^ | 5313 | 5041 | 8283 | 16146 | 18133 | 19219 | 24168 | 15000 | 28460 | 34923 |
| R^2^(L) | 0.9898 | 0.9949 | 0.9849 | 0.9928 | 0.9903 | 0.9904 | 0.994 | 0.9901 | 0.9923 | 0.9943 |
| RF(G)^b^ | 4192 | 4319 | 8095 | 15335 | 19523 | 17706 | 10127 | 7138 | 4219 | 4767 |
| R^2^(G) | 0.9979 | 0.9954 | 0.9945 | 0.998 | 0.9994 | 0.9993 | 0.996 | 0.9921 | 0.9993 | 0.9975 |
| (B) Results of the QA test derived using LWS | | | |  |  |  |  |  |  |  |
| MDL (ng)^c^ | 2.59 | 0.73 | 1.08 | 0.83 | 0.88 | 1.23 | 0.96 | 0.99 | 0.96 | 0.62 |
| MDL (ppb)^d^ | 1.08 | 0.25 | 0.31 | 0.24 | 0.22 | 0.30 | 0.26 | 0.23 | 0.21 | 0.12 |
| RSE (%) | 1.77 | 1.46 | 2.13 | 3.07 | 3.66 | 4.06 | 0.74 | 4.17 | 0.23 | 2.36 |
| ^a^ Response factor (RF) values were derived by ST-based calibration (6 point cal.) using LWS | | | | | | | |  |  |  |
| ^a^ Response factor (RF) values were derived by ST-based calibration (6 point cal.) using GWS prepared in a 1 L PEA bag | | | | | | | | | |  |
| ^c^ To calculate the method detection limit (MDL) values of the target compounds (in ng), the standard deviations (SD) of | | | | | | | | | | |
| seven replicate analyses (1st calibration point LWS) were determined. The MDL values were then calculated as the product | | | | | | | | | | |
| of SD and the student's *t*-value at the 99% confidence level (t=3.14 at 6 degrees of freedom). | | | | | | | |  |  |  |
| ^d^ MDL values were also calculated in ppb units assuming the total volume is 1 L. | | | | | | |  |  |  |  |

**Table 2S.** Relative recovery^a^ (RR_PEA_) of the target compounds in a 1 and 10 L PEA bag

| Order | Variables | ACA | PPA | IBA | BTA | IVA | VLA | PhAl | p-C | ID | SK |
| --- | --- | --- | --- | --- | --- | --- | --- | --- | --- | --- | --- |
| **A. Relative recovery in a 1 L PEA bag** | | |  |  |  |  |  |  |  |  |  |
| RF of LWS (ST-based 6-point calibration by LWS) | | | |  |  |  |  |  |  |  |  |
| 1 | RF_L_ | 5,313 | 5,041 | 8,283 | 16,146 | 18,133 | 19,219 | 24,168 | 15,000 | 28,460 | 34,923 |
| RF of GWS (ST-based 6-point calibration by GWS prepared in a 1 L PEA bag) | | | | | |  |  |  |  |  |  |
| 2 | RF_G_ | 4,192 | 4,319 | 8,095 | 15,335 | 19,523 | 17,706 | 10,127 | 7,138 | 4,219 | 4,767 |
| Relative recovery (%) | |  |  |  |  |  |  |  |  |  |  |
| 3 |  | 78.9 | 85.7 | 97.7 | 95.0 | 108 | 92.1 | 41.9 | 47.6 | 14.8 | 13.7 |
| **B. Relative recovery in 10 L PEA bag** | | | |  |  |  |  |  |  |  |  |
| RF of LWS (ST-based 1-point calibration by LWS) | | | |  |  |  |  |  |  |  |  |
| 1 | RF_L_ | 6,285 | 5,965 | 7,007 | 17,664 | 18,602 | 20,101 | 25,926 | 11,240 | 27,709 | 35,709 |
| RF of GWS (ST-based 1-point calibration by GWS prepared in 10 L PEA bag) | | | | | |  |  |  |  |  |  |
| 2 | RF_G_ | 5,613 | 5,273 | 6,720 | 16,975 | 18,602 | 19,156 | 16,100 | 7,845 | 7,454 | 8,617 |
| Relative recovery (%) | |  |  |  |  |  |  |  |  |  |  |
| 3 |  | 89.3 | 88.4 | 95.9 | 96.1 | 100 | 95.3 | 62.1 | 69.8 | 26.9 | 24.1 |

^a^Relative recovery (RR_PEA_) (%) = (RF_G_/RF_L_) × 100

**Table 3S.** Preparation of liquid working standards (LWS) for analysis by the TD/GC/MS system

**Table 4S.** Operational conditions of TD/GC/MS systems for comparative analysis of the target compounds

| (A). **GC** (Shimadzu GC-2010, Japan)**, MS** (Shimadzu GCMS-QP2010, Japan) | | | |  |
| --- | --- | --- | --- | --- |
| Column: CP Wax (diameter: 0.25 mm, length: 30 m, and film thickness: 0.25 µm) | | | |  |
| *(1) GC Oven settings* | |  | *(2) MS Detector settings* | |
| Oven temp: | 40 °C (5 min) |  | Ionization mode: | EI^b^ (70eV) |
| Oven heating rate: | 20 °C min^-1^ |  | Ion source temp: | 230 °C |
| Max oven temp: | 220 °C (16 min) |  | Interface temp: | 230 °C |
| Total time: | 30 min |  | TIC scan range: | 35-350 m/z |
|  |  |  | Scan speed | 1250 m/z s^-1^ |
| *(3) Carrier gas settings* | |  |  |  |
| Gas type: | He (>99.999%) |  | Initial gas flow: | 1.03 mL min^-1^ |
| Constant gas pressure: | 16.0 psi |  |  |  |
| (B). **Thermal desorber (TD)^a^** | |  |  |  |
| Cold trap sorbent: Quartz wool + Carbopack C + Carbopack B (Volume ratio = 1:1:1) | | | |  |
| Split ratio: | 0.103 |  | Adsorption temp: | 5 °C |
| Split flow: | 10 mL |  | Desorption temp: | 300 °C |
| Trap hold time: | 5 min |  | Flow path temp: | 180 °C |
| (C). **Sorbent (Sampling) Tube** | |  |  |  |
| Sorbent material: | Carbopack C + Carbopack B + Carbopack X (50 mg each) | | |  |
| Desorption flow: | 100 mL min^-1^ |  |  |  |
| Desorption time: | 5 min |  | Desorption temp: | 300 °C |
| ^a^TD (Unity, Markes International, Ltd., UK) | | |  |  |

^b^EI (Electron impact ionization)


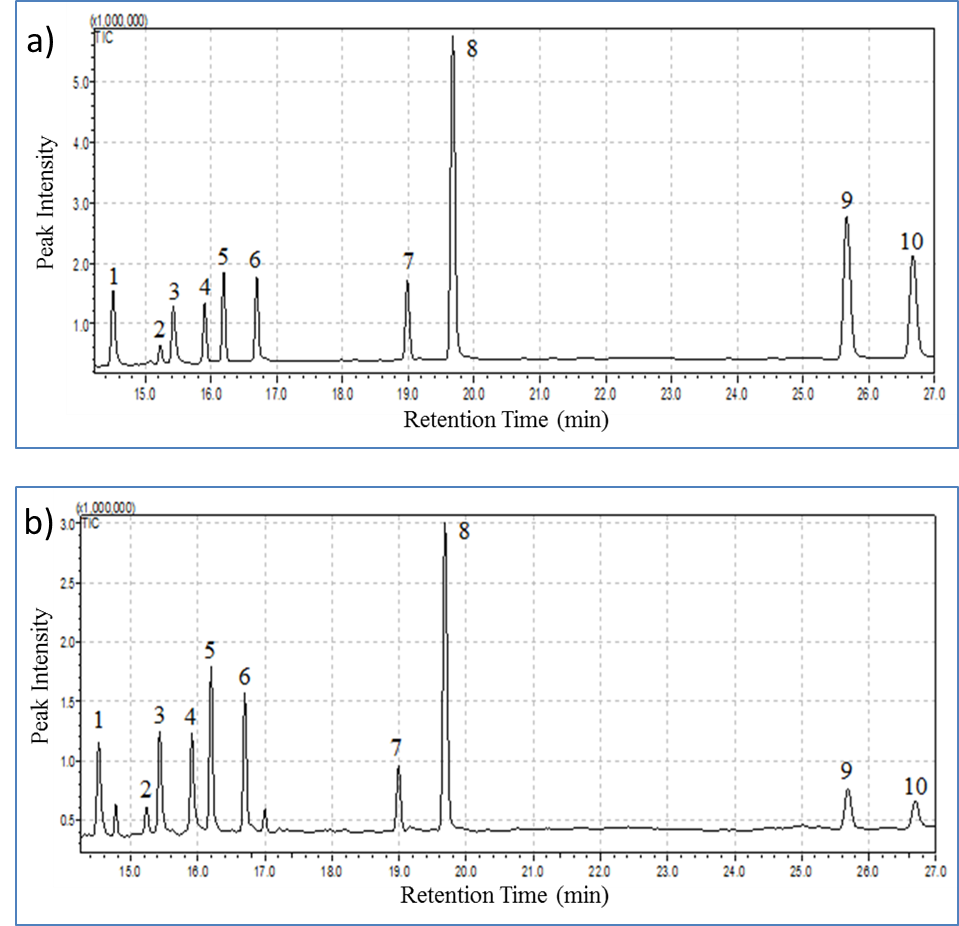


**Figure 1S.** The representative chromatograms of the target compounds obtained from the TD/GC/MS system by direct loading of 1 µL of the 5th calibration point LWS (3a) and 200 mL of vaporized GWS (3b) (same loading mass of LWS) onto the 3-bed sorbent tube: (elution order: (1) Acetic acid, (2) Propionic acid, (3) i-Butyric acid, (4) n-Butyric acid, (5) i-Valeric acid, (6) n-Valeric acid, (7) Phenol, (8) p-Cresol, (9) Indole, and (10) Skatole)


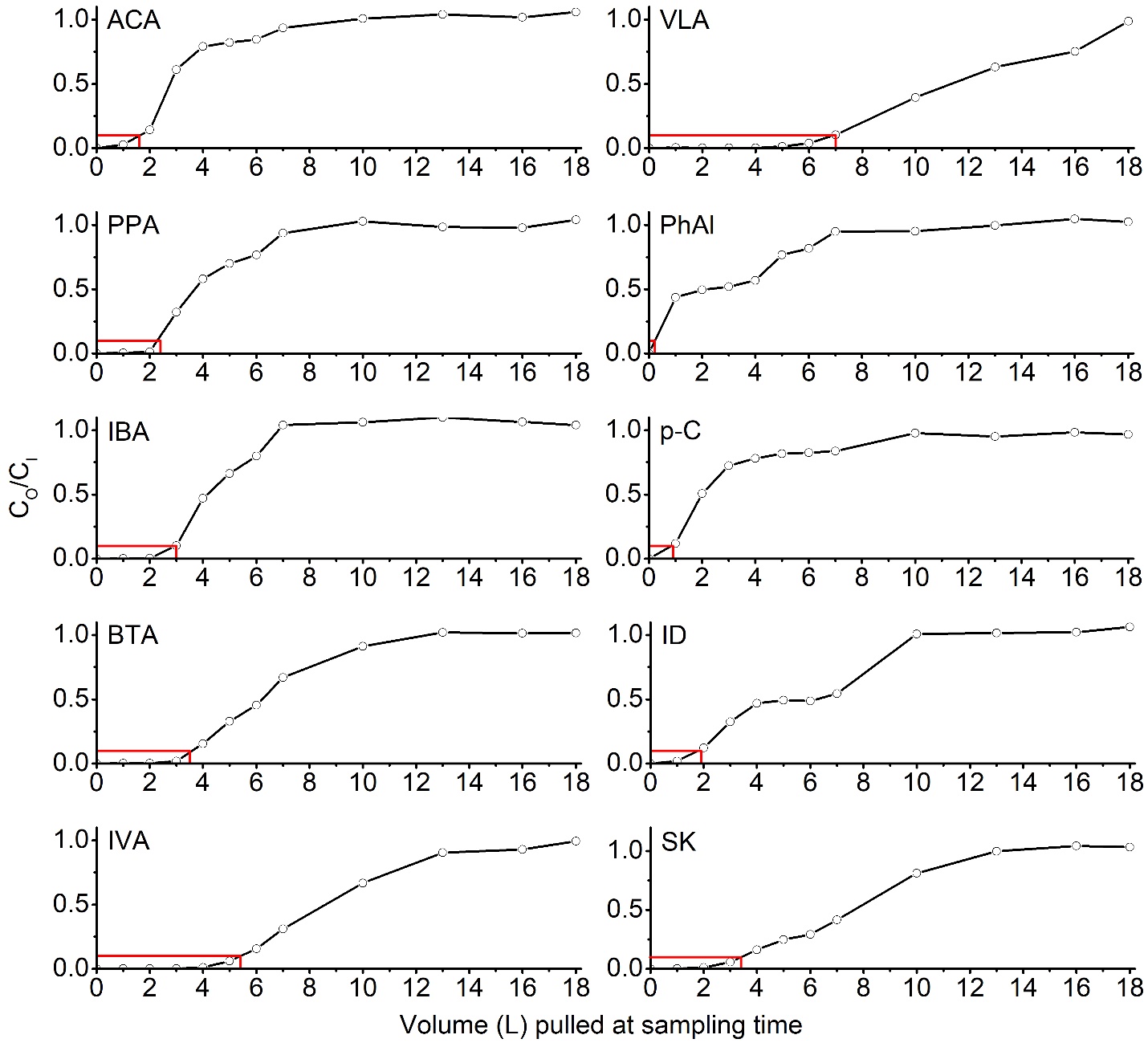


**Figure 2S.** Normalized concentration values required for 173 mL glass impinger (cleaned by method A) saturation by GWS (normalization was made by [C_out_]/[C_in_]) (the red lines indicate 10 % BTV)


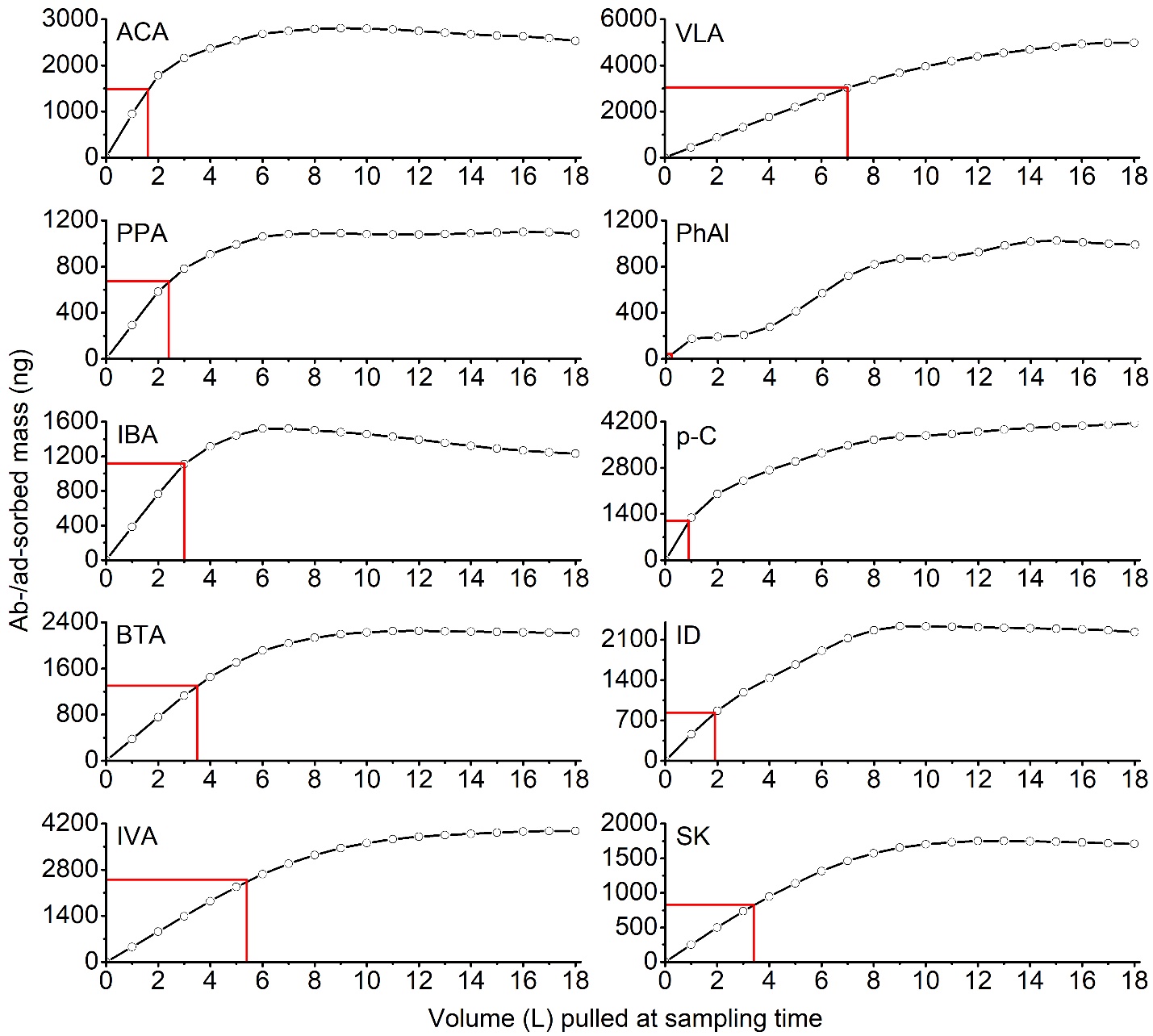


**Figure 3S.** Analyte surface sorption (ng) vs. volume (L) pulled through the 173 mL glass impinger (cleaned by method A) (the red lines indicate 10 % BTV)
